# Comprehensive Analysis of Volatile Flavor Components in ‘Hujing Milu’ Peach from Different Regions Using HS-SPME-GC-MS and HS-GC-IMS

**DOI:** 10.3390/foods15061051

**Published:** 2026-03-17

**Authors:** Yiying Wang, Linshu Jiao, Yiran Gui, Wei Zhao, Lanlan Chen, Xiaolong Chen, Jian Chen, Yong Li, Lixiao Song, Xiangyang Yu

**Affiliations:** 1 Collaborative Innovation Center for Modern Grain Circulation and Safety, College of Food Science and Engineering, Nanjing University of Finance and Economics, Nanjing 210023, China; 1120230497@stu.nufe.edu.cn (Y.W.);; 2 Jiangsu Key Laboratory for Food Quality and Safety-State Key Laboratory Cultivation Base, Ministry of Science and Technology, 50 Zhongling Street, Nanjing 210014, China; 3 Institute of Food Safety and Nutrition, Jiangsu Academy of Agricultural Sciences, 50 Zhongling Street, Nanjing 210014, China; 4 College of Life Science and Food Engineering, Huai’an University, Huai’an 223003, China

**Keywords:** ‘Hujing Milu’ peach, cultivar regions, characteristic volatile compounds, soil physicochemical properties

## Abstract

To explore the characteristic volatile compounds of ‘Hujing Milu’ peaches from different growing regions, headspace solid-phase microextraction coupled with gas chromatography–mass spectrometry (HS-SPME-GC-MS) and headspace gas chromatography–ion mobility spectrometry (HS-GC-IMS) were employed to analyze volatile components in samples from six production areas. A total of 73 and 56 volatile compounds were identified by HS-SPME-GC-MS and HS-GC-IMS, respectively. Quantitative analysis revealed that esters, aldehydes, and alcohols were the main contributors to the aroma profile, accounting for over 70% of the total relative content. Combined with chemometric analysis (VIP > 1 and OAV/ROAV > 1), 17 potential biomarkers were identified that can distinguish ‘Hujing Milu’ peaches from different regions, including ethyl acetate, hexanol, (E)-2-nonenal, and dihydro-β-ionone. Moreover, soil properties of these regions and their correlation with volatile compounds were analyzed to elucidate the formation mechanisms of characteristic aromas. The results showed that ethyl acetate exhibited a significant positive correlation with soil pH (r = 0.530, *p* < 0.05), whereas dihydro-β-ionone showed a significant positive correlation with soil organic matter (r = 0.587, *p* < 0.05) and available potassium (r = 0.830, *p* < 0.05). This study identified characteristic volatile compounds of ‘Hujing Milu’ peaches from different regions, providing a reliable technical basis for origin traceability and the enhancement of aroma quality in ‘Hujing Milu’ peaches.

## 1. Introduction

Peach (*Prunus persica* L.) is an important economic fruit in the Rosaceae family and is widely favored by consumers for its distinctive flavor and rich nutritional value [1,2]. Globally, there are more than 3000 kinds of peach variety resources in the world and more than 1000 kinds of cultivated varieties of peach originated from China [3,4]. ‘Hujing Milu’ peach is well known for its delicate flesh, abundant juiciness, and rich aroma. It is widely cultivated in southern China, including Jiangsu, Zhejiang, Shandong, and Shanghai, yielding considerable economic benefits. In recent years, with consumers’ increasing demands for fruit quality, fruit aroma has been regarded as an important quality characteristic closely related to the commercial value and market competitiveness of fruit [5]. Currently, over 100 volatile compounds have been identified in peach fruits, with only a few key compounds contributing to the aroma profile. The characteristic aroma of peach is primarily composed of esters (hexyl acetate), lactones (γ-decalactone), aldehydes (trans-2-hexenal), and alcohols (cis-3-hexenol) [6,7].

Fruit aroma formation is influenced by multiple factors, including variety, growing environment, climate, and cultivation conditions [8]. Soil conditions, particularly OM content and pH, are key factors affecting fruit aroma quality. These factors influence aroma formation by regulating fruit metabolism, affecting the accumulation of precursors such as sugars, amino acids, and fatty acids, and modulating the activity of enzymes associated with aroma biosynthesis, ultimately contributing to regional quality differences [9,10,11]. Moreover, soil mineral nutrients also play an important role in fruit quality formation. Potassium application has been shown to significantly influence the levels of volatile volatile compounds in cherry tomatoes, including 3-methylbutyraldehyde, phenylacetaldehyde, and phenylethanol [12]. Grape volatile compounds are reported to be more closely related to soil phosphorus and potassium contents than to soil nitrogen content [13]. Growing elevation, temperature, moisture, and light also affect the synthesis of fruit aroma. Falcão et al. investigated the effect of growing temperature on the aroma profiles of ‘Cabernet Sauvignon’ grapes from five vineyards at different altitudes showed that the content of 2-methoxy-3-isobutylpyrazine was significantly positively correlated with vineyard altitude and negatively correlated with temperature [14]. In previous studies, Su et al. indicated that moderate light enhances the synthesis of aromatic compounds in peach fruit, whereas excessive light (full sunlight) or insufficient light (15% of full sunlight) inhibits the accumulation of volatile compounds [15]. Similarly, under greenhouse cultivation conditions, fructose content in apricots increases, but the synthesis of volatile aromas is inhibited due to limited light, resulting in a significant reduction in aroma [16]. These differences in cultivation conditions contribute to the unique flavor profiles, quality attributes, and regional characteristics of fruits from different areas.

At present, research on regional differences in the aroma quality of ‘Hujing Milu’ peaches is limited. To accurately characterize fruit volatile compounds, a combination of detection technologies is necessary to obtain comprehensive and reliable aroma information. HS-SPME-GC-MS is one of the most widely used methods for precise qualitative and quantitative analysis of volatile compounds in fruits [17]. Li et al. analyzed the volatile compounds in three peach cultivars (Jiucui, Zhongyoupan 9, and Zhongyoupan 8) using HS-SPME-GC-MS and identified 169, 159, and 177 volatile compounds, respectively [6]. Gas chromatography–ion mobility spectrometry (GC-IMS) is a relatively new technique for analyzing volatile compounds. It combines the high separation capability of gas chromatography with the high sensitivity of ion mobility spectrometry, enabling rapid collection and analysis of volatile compounds and generation of fingerprint profiles that facilitate intuitive and detailed comparisons among samples [18,19].

To study fruit aroma comprehensively, these methods are often combined to overcome the subjectivity and limited resolution of traditional sensory evaluation, providing a reliable approach for objective analysis of complex volatile compounds [20]. Xu et al. analyzed citrus aromas from different geographical regions using both GC-IMS and HS-SPME-GC-MS and reported that the volatile information obtained by the two methods differed substantially, with GC-MS showing better performance in separating terpenes, alcohols, and esters [21]. Xie et al. applied HS-GC-IMS and HS-SPME-GC-MS to study 10 blueberry cultivars from three regions and identified nine volatile compounds as potential biomarkers for distinguishing varieties and production origins [22].

To comprehensively analyze regional differences in aroma profiles and their formation mechanisms, Hujing Milu’ peach samples were collected from six regions, including Wuxi and Zhangjiagang (Jiangsu Province), Fenghua (Zhejiang Province), Fengxian (Shanghai), Jianyang (Sichuan Province), and Mengyin (Shandong Province). The chosen study regions were well-known production areas for honey peaches including ‘Hujing Milu’ characterized by a long history of cultivation and relatively high yields. These regions covered major production areas in eastern, southeastern, and southwestern China, which facilitated a systematic study regional difference. HS-SPME-GC-MS and HS-GC-IMS were employed to detect volatile compounds in the peaches, and characteristic volatiles in ‘Hujing Milu’ peaches from different regions were screened using principal component analysis (PCA) and orthogonal partial least squares discriminant analysis (OPLS-DA). Furthermore, differences in volatile compounds among regions and their correlation with soil quality were examined. This study aims to reveal the regional variation characteristics of volatile compounds in ‘Hujing Milu’ peaches and clarify the relationship between soil properties and aroma formation, thereby providing a theoretical foundation for the origin identification and quality improvement of this specialty peach variety.

## 2. Materials and Methods

### 2.1. Sample Collection and Pretreatment

‘Hujing Milu’ peaches were collected from six producing areas: Wuxi and Zhangjiagang in Jiangsu Province, Fenghua in Zhejiang Province, Fengxian in Shanghai, Jianyang in Sichuan Province, and Mengyin in Shandong Province. In each orchard, five sampling points were selected, and peach trees at the same growth stage were chosen for sampling. Fruits were randomly harvested from different positions on each tree (top, middle, and bottom). Six fruits were collected at each sampling point, resulting in 30 fruits per orchard as one batch. All samples were immediately placed in foam boxes with ice packs and transported to the laboratory. Undamaged fruits with similar color and size were selected, peeled, cut into pieces, and stored at −80 °C for subsequent analysis.

Soil samples were collected from the same locations as the peach samples. At each planting base, soil was collected from five sampling points. A total of 300 g of soil was obtained per batch, with 60 g taken from each sampling point. After removing debris such as plant and animal residues, the soil samples were spread into a thin layer of 2–3 cm and allowed to air-dry naturally in a cool environment. The dried soil samples were then crushed using a wooden hammer, further pulverized using a wooden stick, and subsequently sieved through nylon sieves with pore sizes of 2 mm, 0.25 mm, and 0.149 mm. Finally, the samples were stored separately according to their particle size for subsequent analysis [23,24].

### 2.2. Analysis of Volatile Compounds in Peach Fruits

#### 2.2.1. HS-SPME-GC-MS Analysis of Volatile Compounds

Volatile compounds in ‘Hujing Milu’ peaches were analyzed using an HS-SPME-GC-MS system (Agilent 8890–7000, Santa Clara, CA, USA). The sample extraction procedure was modified from Cai et al. [10]. Briefly, 3 g of ground peach sample were placed into a 20 mL headspace vial containing a magnetic stir bar, followed by the addition of 3 mL saturated NaCl solution and 30 μL of 3-nonanone as an internal standard. The vials were equilibrated on a thermostatically controlled magnetic stirrer at 40 °C with a stirring rate of 600 r/min for 30 min. Subsequently, a solid phase microextraction fiber (65 μm PDMS/DVB) was exposed to the headspace for adsorption for 30 min. After extraction, the fiber was inserted into the GC injector for desorption for 5 min.

GC-MS conditions were set according to Otify et al. [25]. GC separation was performed using an HP-5MS capillary column (60 m × 0.25 mm, 0.25 μm). The inlet temperature was set at 250 °C, and helium was used as the carrier gas at a flow rate of 1 mL/min in splitless injection mode. The oven temperature program was as follows: the initial temperature was held at 35 °C for 2 min; then increased to 220 °C at a rate of 4 °C/min and held for 2 min; and finally increased to 245 °C at a rate of 15 °C/min. The MS conditions were set with an electron ionization (EI) source at 70 eV and a mass range of 35–350 *m*/*z*. The transfer line temperature, ion source temperature, and quadrupole temperature were set at 280 °C, 250 °C, and 150 °C, respectively.

Under the same experimental conditions, GC-MS was used to analyze saturated alkane standards (C_7_–C_40_) to determine the retention index (RI). The RI values of the volatile components were calculated using linear interpolation based on the retention times of n-alkanes according to the following formula [26]:

(1) *RI* = 100 × *n* + 100 × (*t_a_* − *t_n_*)/(*t_n_*_+1_ − *t_n_*)

where *n* represented the number of carbon atoms, while *t_a_*, *t_n_*, and *t_n+_*_1_ represented the retention times of the volatile compound, *C_n_*, and *C_n_*_+1_, respectively.

The calculated RI values of each volatile component were compared with the RI values in the NIST 17 database, and identification was performed by matching both the RI values and the mass spectra with the standard mass spectra. The identified volatile components with matching factors higher than 70% were considered reliable. In addition, 3-nonanone was used as an internal standard for the relative quantification analysis of volatile components in the peach samples.

#### 2.2.2. HS-GC-IMS Analysis of Volatile Compounds

Volatile compounds in peach samples were also analyzed using a GC-IMS instrument (FlavorSpec^®^, G.A.S., Dortmund, Germany). Two g of peach fruit powder were placed into a 20 mL headspace vial and incubated at 40 °C for 15 min. The headspace injection needle temperature was set at 85 °C, and the injection volume was 500 μL.

GC conditions followed Xu et al. [27]. An MXT-5 capillary column (15 m × 0.53 mm, 1 μm) was used for chromatographic separation, with an initial column temperature of 60 °C. High-purity nitrogen (≥99.99%) served as the carrier gas, and the flow rate program was set as follows: 2 mL/min for 2 min, increased to 10 mL/min for 8 min, and then increased to 100 mL/min for 10 min. IMS conditions were as follows: drift tube length 9.8 cm, drift tube temperature 45 °C, and electric field strength 500 V/cm. High-purity nitrogen (≥99.99%) was used as the drift gas at a flow rate of 150 mL/min.

A calibration curve between retention time (RT) and retention index (RI) was established using six n-ketone mixed standards. Volatile compounds were identified by matching both RI and ion mobility drift time with reference data from the GC-IMS built-in library and the NIST database. Flavor fingerprints of volatile compounds were constructed using the reporter plot and gallery plot modules in the VOCal software (v 0.4.03).

#### 2.2.3. Calculation of Odor Activity Value (OAV) and Relative Odor Activity Value (ROAV)

To evaluate the contribution of individual volatile compounds to the overall aroma, the odor activity value (OAV, Formula (2)) and relative odor activity value (ROAV, Formula (3)) were calculated. Higher OAV or ROAV values indicate greater contributions to the total aroma [28].

(2) *OAV* = *C*/*OT*

where *C* represented the concentration of a volatile compound, and *OT* was its odor threshold in water (μg/kg). Odor threshold values were obtained from *Compilation of Odor Thresholds in Air, Water, and Other Media* [29].

(3) *ROAV_a_* (%) = *C_a_*/*C_max_* × *T_max_*/*T_a_* × 100

where *C_a_* was the relative content of volatile compounds (%); *T_a_* was the olfactory threshold for volatile compounds (μg/kg); *C_max_* and *T_max_* represented the relative content (%) and odor threshold (μg/kg) of the compound that contributes most significantly to the overall aroma of the sample.

### 2.3. Determination of Soil Physicochemical Properties

The soil samples were collected and prepared using the five-point sampling method in Section 2.1 to the determination of physicochemical indicators. Soil organic matter (OM) content was determined using the potassium dichromate oxidation–volume method [30]. Available phosphorus (AP) was measured using the sodium bicarbonate extraction–molybdenum antimony colorimetric method [31]. Soil available potassium (AK) was determined by flame photometry [32]. Soil pH was measured using a pH meter [33].

### 2.4. Data Analysis

Each experiment was conducted in triplicate, and the results were expressed as the mean ± standard deviation (SD). Excel 2021 was used for data organization. SPSS 22.0 was used to perform one-way analysis of variance (ANOVA) and normality tests, followed by Tukey–Kramer post hoc tests after ANOVA. Data normalization and graphical visualization were conducted using Origin 2024. PCA and partial least squares discriminant analysis (PLS-DA) were performed using the MetaboAnalyst online platform (https://www.metaboanalyst.ca/, accessed on 31 October 2025). Pearson correlation analysis and heatmap visualization were conducted using the Metware Cloud online tool (https://cloud.metware.cn/, accessed on 13 November 2025).

## 3. Results and Discussion

### 3.1. HS-SPME-GC-MS Analysis of Volatile Compounds in Different Regions of ‘Hujing Milu’ Peach

#### 3.1.1. Differences in Volatile Compounds of ‘Hujing Milu’ Peaches from Different Regions Based on HS-SPME-GC-MS

**Figure 1:**
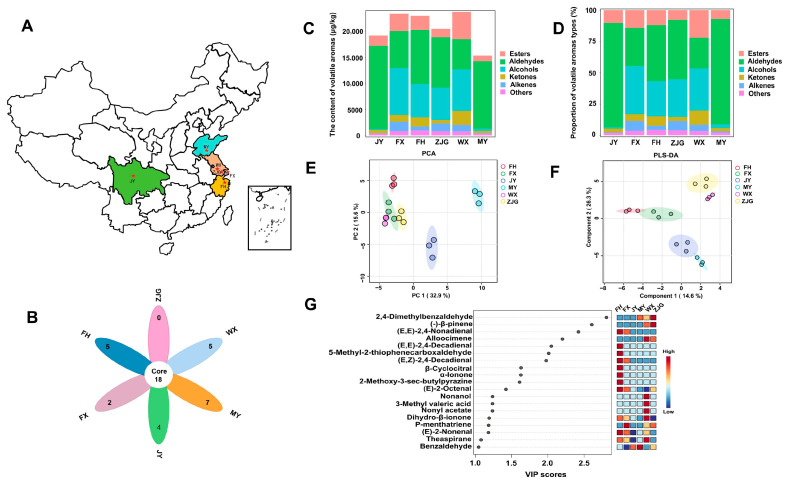
Comparison of volatile compounds in ‘Hujing Milu’ peaches from different regions based on HS-SPME-GC-MS. (**A**) Sampling locations of ‘Hujing Milu’ peach samples; (**B**) Venn diagram of volatile compounds; (**C**) Total volatile compounds content; (**D**) Proportion of volatile compound classes; (**E**) PCA of volatile compounds; (**F**) PLS-DA of volatile compounds; (**G**) VIP values and content heatmap of volatile compounds.

Fruit aroma is a crucial indicator for evaluating fruit quality and is mainly composed of aldehydes, alcohols, esters, terpenes, and ketones, which are derived from amino acids, carotenoids, and fatty acids [34]. HS-SPME-GC-MS is a widely used method for determining volatile compounds in fruits. In this study, the volatile compounds of ‘Hujing Milu’ peaches from six different regions were analyzed using HS-SPME-GC-MS. Based on a matching factor (≥70%), a total of 73 volatile compounds were identified, as detailed in Table 1. This includes 17 esters, 17 aldehydes, 11 ketones, 11 alkenes, 7 alcohols, and 11 other compounds. The concentrations and relative contents of individual volatile compounds in the samples are shown in Figure 1. Aldehydes and alcohols were found to be the predominant volatile compounds in ‘Hujing Milu’ peaches, followed by esters. This result is consistent with the findings of Wu et al., who reported that alcohols, aldehydes, and esters were the aroma classes with the highest relative contents in seven peach cultivars analyzed using HS-SPME-GC-MS [35]. Similarly, Farneti et al. identified aldehydes, alcohols, terpenoids, and esters as the main compound classes contributing to the aroma profiles of blueberries [36].

Among the 73 volatile compounds identified in this study, 18 were common across all six regions, including 2-hexenal, benzaldehyde, γ-decalactone, and dihydro-β-ionone, which play key roles in the aroma of ‘Hujing Milu’ peaches (Figure 1B). The typical aroma classes commonly found in ‘Hujing Milu’ peaches were aldehydes, esters, and ketones, which exhibited marked differences in their contributions to regional aroma profiles. Aldehydes such as hexanal and 2-hexenal are major contributors to green notes in fruits [37,38], and benzaldehyde has been reported as a dominant aldehyde in peach pulp [39,40]. Esters are formed through esterification reactions between short-chain fatty acids and alcohols. As major contributors to fruity aromas, esters strongly affect the overall aroma profile of peaches [41,42], particularly lactones, which contribute characteristic fruity and sweet notes due to their low odor thresholds [5,43]. Ketones are mainly generated through pathways such as fatty acid oxidation, carotenoid degradation, and decarboxylation, typically imparting floral notes [5,44].

The volatile compounds of ‘Hujing Milu’ peaches from different regions are illustrated in Figure 1C,D. The results showed that the total aroma content was highest in samples from the WX region (23,710.90 μg/kg), followed by samples from the FX (23,317.92 μg/kg) and FH (22,935.44 μg/kg) regions. Notably, the proportion of aldehydes in samples from the MY and JY regions was significantly higher than that in other regions, reaching 84.16% and 83.23%, respectively. In contrast, the proportions of alcohols in MY and JY samples were relatively low, accounting for only 2.91% and 0.92%, respectively, which differed significantly from those in other regions. Samples from the WX region display comparatively high proportions of esters (22.07%) and ketones (11.44%), while samples from the FX region displayed distinct regional characteristics with a high proportion of alcohols (38.34%). Li et al. analyzed volatile compounds of peaches from three different regions (Beijing, Shandong, and Hebei) and reported significant regional differences in the proportions of aldehydes, alcohols, esters, and ketones [45]. Similarly, Xiao et al. investigated aroma characteristics of ‘Jinxiu’ yellow peaches from three regions and demonstrated that aldehydes were the dominant aroma component [46].

#### 3.1.2. Multivariate Statistical Analysis of Volatile Compounds of ‘Hujing Milu’ Peach from Different Regions Based on HS-SPME-GC-MS

To characterize the overall differences in the aroma profiles of ‘Hujing Milu’ peaches from different geographical regions, principal component analysis (PCA) was conducted using aroma component data from the six regions. As illustrated in Figure 1**E**, the contribution rates of PC1 and PC2 were 32.9% and 15.6%, respectively, resulting in a cumulative contribution rate of 48.5%. This result effectively explains the major variation in overall aroma among ‘Hujing Milu’ peach samples from the six regions. Based on clustering patterns in the PCA score plot, samples from the WX, ZJG, FX, and FH regions were closely clustered, indicating a high degree of similarity in their overall aroma profiles. In contrast, samples from the MY region showed clear spatial separation from those of other regions, indicating a distinctly different aroma profile, which is consistent with its greater geographical separation.

To further analyze characteristic volatile compounds of ‘Hujing Milu’ peaches from different regions, partial least squares discriminant analysis (PLS-DA) was employed for dimensionality reduction. The reliability of the PLS-DA model was verified using five-fold cross-validation (Figure S1). The R^2^ and Q^2^ values were 0.975 and 0.853, respectively, indicating good model robustness and predictive performance. As shown in Figure 1F, the contribution rates of Component 1 and Component 2 were 14.6% and 26.3%, respectively, resulting in cumulative contribution rate of 40.9%, which adequately explains aroma differences among samples. The PLS-DA score plot showed that WX and ZJG clustered together, as did FH and FX, while clear separations were observed between WX and ZJG, FH and FX, and MY and JY. Variable importance in projection (VIP) values were calculated to evaluate the contribution of each aroma compound to the model, with higher VIP values indicating greater contributions [47]. A total of 19 compounds with VIP > 1 were identified (Figure 1G), indicating their key roles in differentiating aroma profiles among regions. The corresponding heatmap shows relative concentrations of these compounds. Ranked by VIP values, the major differential compounds contributing to aroma differences included 2,4-dimethylbenzaldehyde, (−)-β-pinene, (E, E)-2,4-nonadienal, allo-ocimene, (E, E)-2,4-decadienal, 5-methyl-2-thiophenecarboxaldehyde, (E, Z)-2,4-decadienal, β-cyclocitral, α-ionone, 2-methoxy-3-sec-butylpyrazine, (E)-2-octenal, nonanol, 3-methylvaleric acid, nonyl acetate, dihydro-β-ionone, p-menthatriene, (E)-2-nonenal, theaspirane, and benzaldehyde. Among these compounds, benzaldehyde and dihydro-β-ionone have been reported as core volatile compounds of peaches, and their regional concentration differences are key factors contributing to the different aroma characteristics of ‘Hujing Milu’ peaches [45,48].

#### 3.1.3. Screening of Characteristic Volatile Compounds of ‘Hujing Milu’ Peach from Different Regions Based on HS-SPME-GC-MS

The overall aroma profile of peaches is influenced by multiple factors. Compounds present at high concentrations establish the foundational structure of aroma, while certain compounds present at low concentrations but with low odor thresholds also significantly shape aroma characteristics [49]. The OAV is a quantitative indicator used to evaluate the contribution of individual volatile compounds to the overall aroma profile. Compounds with OAV > 1 are generally considered key contributors [28]. To further investigate the contribution of volatile compounds, OAV analysis was performed for the 73 volatile compounds detected by HS-SPME-GC-MS, and 48 compounds with OAV > 1 were identified. Among these, samples from the six producing regions (JY, FX, FH, ZJG, WX, and MY) contained 21, 34, 33, 28, 30, and 21 compounds, respectively (Table S1).

In addition, 17 volatile compounds with high contributions (OAV > 100) were identified, including γ-decalactone, γ-dodecalactone, (E)-2-nonenal, (E)-2-octenal, 2-hexenal, (E)-2-decenal, (E, E)-2,4-nonadienal, nonanal, (E, Z)-2,4-decadienal, (E, E)-2,4-decadienal, linalool, 1-hepten-3-one, dihydro-β-ionone, (E)-β-ionone, β-damascenone, myrcene, and 2-methoxy-3-sec-butylpyrazine. Among these, γ-decalactone, γ-dodecalactone, 2-hexenal, linalool, and dihydro-β-ionone have been widely reported as key contributors to peach aroma, with γ-decalactone providing a characteristic fruity and peach-like note [50]. Notably, concentrations of γ-decalactone, nonanal, dihydro-β-ionone, and β-damascenone, all with OAV > 100, were significantly higher in samples from five regions (JY, FX, FH, ZJG, and WX) compared to samples from the MY region, indicating stronger fruity and floral aroma characteristics.

Clear differences in characteristic aromas were also observed among regions. In samples from the JY region, β-damascenone and 1-hepten-3-one showed the highest OAVs, contributing predominantly floral notes. In samples from the FX and ZJG regions, linalool and β-damascenone were the main contributors, resulting in pronounced fruity and floral characteristics. In samples from the WX region, linalool and (E)-β-ionone exhibited the highest OAVs, contributing sweet floral and fruity notes. In samples from the FH region, linalool and 2-methoxy-3-sec-butylpyrazine were identified as major contributors, producing a combined aroma characterized by floral, fruity, green, and slight frankincense notes. In contrast, samples from the MY region were characterized by 1-hepten-3-one and (E)-2-nonenal as dominant components, imparting a fresh green aroma.

To further screen differential aroma markers of ‘Hujing Milu’ peaches from different regions, a dual screening criterion of OAV > 1 and VIP > 1 was applied. A total of 12 differential volatile compounds were identified (Table 2), including (E)-2-nonenal, (E)-2-octenal, benzaldehyde, (E, E)-2,4-nonadienal, dihydro-β-ionone, and 2-methoxy-3-sec-butylpyrazine. Notably, (E)-2-nonenal and (E)-2-octenal were key markers distinguishing samples from the FH region, contributing rich fruity and frankincense-like notes. In samples from the WX region, dihydro-β-ionone showed the highest OAV and was the primary source of intense floral aroma, whereas benzaldehyde significantly contributed to aroma characteristics of samples from the MY region.

### 3.2. HS-GC-IMS Analysis of Volatile Compounds in Different Regions of ‘Hujing Milu’ Peach

#### 3.2.1. Separation, Characterization, and Comparative Analysis of Volatile Compounds of ‘Hujing Milu’ Peaches from Different Regions Based on HS-GC-IMS

**Figure 2:**
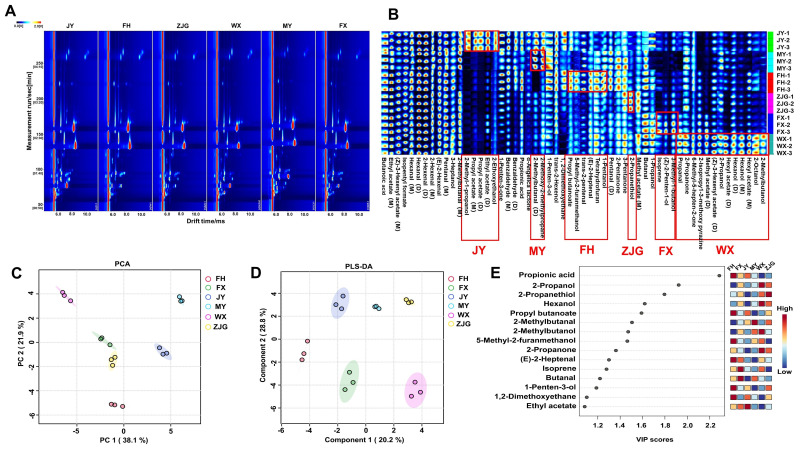
Comparison of volatile compounds in ‘Hujing Milu’ peaches from different regions based on HS-GC-IMS. (**A**) Topographic plots of ‘Hujing Milu’ peaches from different regions. The ordinate represents gas chromatography retention time, and the abscissa represents ion mobility drift time. The red vertical line at coordinate 1.0 indicates the normalized reactant ion peak (RIP). (**B**) Aroma fingerprints of ‘Hujing Milu’ peaches from different producing areas. Each row represents one volatile compound across different samples, and each column represents signal peaks of a single sample. Relative concentration of volatile compounds is indicated by color intensity, with brighter colors corresponding to higher concentrations. Red boxes highlight volatile compounds with relatively high concentrations in peach samples from different producing areas. (**C**) PCA of volatile compounds; (**D**) PLS-DA of volatile compounds; (**E**) VIP values and content heatmap of volatile compounds.

HS-GC-IMS is a recently developed gas-phase separation and detection technique. Its main advantage is the combination of the high separation capability of gas chromatography with the rapid response of ion mobility spectrometry. Compared to HS-SPME-GC-MS, HS-GC-IMS is characterized by high separation efficiency, rapid analysis, and operation under atmospheric pressure and moderate temperature conditions [51,52]. To further investigate differences in volatile compound composition of ‘Hujing Milu’ peaches from different producing areas, HS-GC-IMS was applied to obtain aroma information from peach samples of different origins. Two-dimensional visualization of the detected volatile compounds is shown in Figure 2A. A total of 56 volatile compounds were identified, which included 15 aldehydes, 15 alcohols, 13 esters, 5 ketones, and 8 other compounds (Table 3). Aldehydes, alcohols, and esters were the dominant volatile compounds in peaches from all six regions, consistent with the results obtained using HS-SPME-GC-MS. Some volatile compounds with high proton affinity tend to form dimers or polymers through proton or electron sharing, leading to multiple signal peaks. In this study, monomers and dimers of these compounds are denoted as (M) and (D), respectively [53,54].

To accurately evaluate regional differences in volatile compounds of ‘Hujing Milu’ peaches, aroma fingerprints of samples from the six regions were constructed (Figure 2B), followed by semiquantitative analysis. The results showed that 3-heptanol, pentanal (D), (E)-2-hexenal, 2-hexenal (M), 2-hexenal (D), hexanal (M), hexanal (D), isopentyl formate, (Z)-3-hexenyl acetate (D), ethyl acetate (M), and butanoic acid were present at relatively high levels in ‘Hujing Milu’ peach samples. Notably, samples from the JY region exhibited significant differences in five volatile compounds, including ethyl acetate (D), propyl acetate and 2-methyl-1-propanol, when compared to samples from other regions. In the MY region, contents of 2-methylbutanal (D) and 2-methoxy-2-methylpropane were significantly higher than those in the other five regions. The FH region samples contained six volatile compounds, including 1-pentanol, tetrahydrofuran, (E)-2-heptenal, trans-2-pentenal, 5-methyl-2-furfuryl alcohol and propyl butanoate, which were detected at relatively high levels. In the ZJG region, the content of 2-propanethiol was higher than in samples from other regions. Furthermore, in the FX region, three volatile compounds, including 3-methyl-1-butanol, (Z)-2-pentenol and isoprene were present at notably high levels. Compared with the other five regions, 14 volatile compounds were detected in samples from the WX region (Figure 2B). Among these, 2-methylbutanol, hexyl acetate and 2-octanol were present at relatively high levels. Both analytical techniques identified hexyl acetate and 2-hexenal. The content of hexyl acetate is positively correlated with the fruity and sweet aroma of the fruit, enhancing fruity intensity in fruits [55].

A comparative analysis of HS-GC-IMS and HS-SPME-GC-MS results was further conducted. HS-SPME-GC-MS demonstrated superior separation capability for volatile compounds compared to HS-GC-IMS. However, HS-GC-IMS has distinct advantages in detecting small-molecule and low-abundance volatile compounds, such as ketones and acids [56,57]. In addition, HS-GC-IMS identified 33 compounds that were not detected by HS-SPME-GC-MS, including butanal with a cocoa-like note, hexanal with a fatty note, 2-propanol with a woody note, propyl butanoate with a pineapple-like note, and 2-pentanone with a fruity note. These findings indicated that HS-GC-IMS effectively complements HS-SPME-GC-MS, facilitating a comprehensive analysis of volatile compounds. The combined application of HS-GC-IMS and HS-SPME-GC-MS integrates the advantages of strong separation capability, high sensitivity, and reliable qualitative and quantitative performance, thereby expanding the detectable range of volatile compounds, and providing a more comprehensive representation of fruit flavor characteristics [58].

#### 3.2.2. Multivariate Statistical Analysis of Volatile Compounds of ‘Hujing Milu’ Peach from Different Regions Based on HS-GC-IMS

Principal component analysis (PCA) of volatile compounds in ‘Hujing Milu’ peaches from different regions was conducted, with the results are shown in Figure 2C. The contribution rates of principal components PC1 and PC2 were found to be 38.1% and 21.9%, respectively, resulting in a cumulative contribution rate of 60.0%. This indicates that the PCA model effectively characterizes overall aroma differences among ‘Hujing Milu’ peach samples from different geographical origins. In the PCA score plot, samples from the ZJG and FX regions were closely clustered, indicating high similarity in their overall aroma profiles. This similarity may be related to comparable climatic conditions, such as temperature and light, or similar soil physicochemical properties in the two regions. In contrast, samples from the MY, JY, WX, and FH regions were clearly separated from the ZJG–FX cluster, indicating relatively significant differences in overall aroma characteristics.

Notably, compared to results obtained using HS-SPME-GC-MS, the combination of HS-GC-IMS with PCA showed stronger discriminative performance in characterizing aroma differences among samples from different geographical origins. This observation is consistent with the findings of Zhang et al. in citrus volatile analysis, which demonstrated that HS-GC-IMS combined with multivariate analysis provides improved resolution for distinguishing geographical origins of agricultural products [59]. He et al. also reported the advantages of GC-IMS in identifying differences in volatile components and discriminating geographical origins of kiwifruit [56].

Partial least squares discriminant analysis (PLS-DA) was conducted to identify volatile compounds that most significantly contribute to differences in overall aroma profiles. The PLS-DA model was validated using five-fold cross-validation, yielding R^2^ and Q^2^ values of 0.98937 and 0.96207, respectively (Figure S2), indicating strong model reliability and predictive capability. As shown in Figure 2D, the contribution rates of Component 1 and Component 2 were 20.2% and 28.8%, respectively, with a cumulative contribution rate of 49.0%. The PLS-DA score plot showed clear separation of ‘Hujing Milu’ peach samples from the six regions, confirming that geographical origin is a major factor associated with aroma differences. This finding is consistent with the conclusions reported by Li et al. regarding the correlation between peach volatile compounds and geographical origin [45]. Variable importance in projection (VIP) values were calculated, and 15 differential volatile compounds with VIP > 1 were identified (Figure 2D). These compounds played key roles in differentiating aroma profiles among regions and were ranked by VIP value as follows: propionic acid, 2-propanol, 2-propanethiol, hexanol, propyl butanoate, 2-methylbutanal, 2-methylbutanol, 5-methyl-2-furanmethanol, 2-propanone, (E)-2-heptenal, isoprene, butanal, 1-penten-3-ol, 1,2-dimethoxyethane, and ethyl acetate. Among these, ester compounds such as ethyl acetate have been reported as characteristic aroma contributors in peaches, and differences in their contents directly affect peach aroma characteristics. This observation is consistent with the results reported by Li et al. during screening of key peach volatile compounds [45].

#### 3.2.3. Screening of Characteristic Volatile Compounds of ‘Hujing Milu’ Peach from Different Regions Based on HS-GC-IMS

To evaluate the contribution of individual volatile compounds to the overall aroma of ‘Hujing Milu’ peaches, the relative ROAV of the 56 volatile compounds identified by HS-GC-IMS was calculated. Compounds with ROAV ≥ 1 were defined as potential key aroma contributors. A total of 13 volatile compounds met this criterion (Table S2), including (Z)-3-hexenyl acetate, hexyl acetate, ethyl acetate, propyl butanoate, hexanal, 2-hexenal, 2-methylbutanal, butanal, pentanal, hexanol, 3-methyl-1-butanol, 2-octanol, and 2-methoxy-2-methylpropane. Hexanal showed the highest contribution (ROAV = 100) in samples from the JY, FX, FH, WX, and ZJG regions and the second highest contribution (ROAV = 97.82) in samples from the MY region, indicating that hexanal is a major aroma-active compound in ‘Hujing Milu’ peaches. Tan et al. reported that hexanal, because of its low odor threshold and strong olfactory activity, frequently serves as a core aroma compound in flat peach juice, which supports its key role in the aroma profile of ‘Hujing Milu’ peaches [60].

To identify characteristic volatile compounds responsible for regional aroma differences, volatile compounds were further screened using combined criteria of ROAV > 1 and VIP > 1. Five volatile compounds meeting these criteria were identified (Table 4): ethyl acetate, propyl butanoate, 2-methylbutanal, butanal, and hexanol. In samples from the JY region, ethyl acetate showed a significantly higher ROAV compared to other regions, indicating its value as a biomarker for distinguishing JY samples and contributing to an intensified fruity aroma. In samples from the FH region, propyl butanoate exhibited the highest ROAV among all regions, contributing a sweet and fresh pineapple-like aroma. In samples from the WX region, hexanol showed the highest ROAV, contributing a combined fruity and green aroma profile. In samples from the MY region, 2-methylbutanal significantly contributed to the aroma, imparting a characteristic cocoa-like note.

Based on integrated analyses using OAV, ROAV, and PLS-DA, characteristic volatile compounds of ‘Hujing Milu’ peaches from six regions were identified (Table 5). In the JY region, the main characteristic volatile compounds were benzaldehyde and hexanol. In the FX region, the identified characteristic volatile compounds included (E, Z)-2,4-decadienal, (E, E)-2,4-nonadienal, p-menthatriene, and butanal. In the FH region, characteristic volatile compounds included 2-methoxy-3-sec-butylpyrazine, (E)-2-nonenal, (E, E)-2,4-decadienal, ethyl acetate, (E)-2-octenal, β-cyclocitral, 5-methylthiophene-2-carboxaldehyde, (E, Z)-2,4-decadienal, and α-ionone. In the ZJG region, characteristic volatile compounds included propyl butanoate and p-menthatriene. In the WX region, characteristic volatile compounds included dihydro-β-ionone and p-menthatriene. Finally, in the MY region, characteristic volatile compounds were benzaldehyde and 2-methylbutanal.

### 3.3. Correlation Analysis Between Volatile Compounds and Cultivation Environments of ‘Hujing Milu’ Peaches from Different Origins

**Figure 3:**
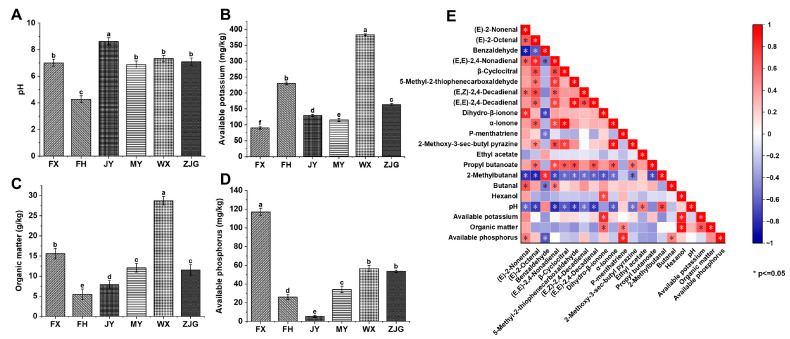
(**A**) pH; (**B**) Available potassium; (**C**) Organic matter; (**D**) Available phosphorus; (**E**) Correlation analysis between characteristic volatile compounds of ‘Hujing Milu’ peaches and soil physico-chemical properties from different regions (* indicates significance at *p* ≤ 0.05). Different letters indicate significant differences (*p* < 0.05), the error bars represented SD.

Soil serves as an essential environmental carrier for plant growth. The environmental differences in terrain and climate among cultivation regions are ultimately reflected in soil physicochemical properties, including pH, AK, AP, and OM. Consequently, soil properties act as a key intermediate through which regional environmental conditions affect fruit aroma [61,62]. Soil physicochemical properties of six ‘Hujing Milu’ peach production regions were determined, and the results are shown in Figure 3A–D. Significant differences (*p* < 0.05) were observed among regions for all measured indicators (pH, AK, OM, and AP).

The JY region exhibited the highest soil pH value (8.62), while the FH region showed the lowest pH value (4.28). Soil pH values in the FX, MY, WX, and ZJG regions ranged from 6 to 8, with no significant differences among these regions. Significant regional differences were observed in AK content, which decreased in the order WX (382.90 mg/kg), FH (230.10 mg/kg), ZJG (163.20 mg/kg), JY (129.90 mg/kg), MY (115.50 mg/kg), and FX (89.76 mg/kg). The OM content in the WX region (28.77 g/kg) was also significantly higher than in other regions and was nearly fivefold higher than that in the FH region (5.50 g/kg). The FX region had the highest AP content (117.10 mg/kg), which was significantly greater than that in all other regions. In contrast, the JY region had the lowest AP content (5.53 mg/kg), with significant variations observed in the remaining regions. These results indicate clear regional specificity in soil physicochemical properties, which may indirectly affect the synthesis and accumulation of volatile compounds [63,64]. Soil factors can influence volatile aroma formation through multiple pathways, including regulation of plant physiological metabolism, microbial activity, and enzyme activity [11,65]. Ji et al. reported that the application of organic fertilizer application significantly improved soil pH and OM content while altering microbial diversity, with microbial interactions having strong effects on volatile emissions [66]. Ran et al. showed that OM content affects soil structure, water retention, and nutrient buffering capacity, thereby enhancing metabolic activity of tea plants and regulating the synthesis of secondary metabolites related to flavor and aroma [67].

To clarify the correlation between soil conditions and aroma formation in ‘Hujing Milu’ peaches, Pearson correlation analysis was conducted to systematically examine associations between soil physicochemical properties and concentrations of characteristic volatile compounds. As shown in Figure 3E, most aldehydes exhibited significant negative correlations with soil pH. However, 2-methylbutanal showed a significant positive correlation with soil pH (r = 0.690, *p* < 0.05). In addition, ethyl acetate content was positively correlated with soil pH (r = 0.530, *p* < 0.05), indicating that alkaline soil conditions favor its synthesis. This effect may be related to enhanced lipoxygenase activity under alkaline conditions, which can accelerate ethyl acetate formation [68,69]. Wang et al. reported that increased soil pH promotes synthesis of floral volatile compounds in tea, particularly geraniol, thereby enhancing floral characteristics in the overall aroma profile [62].

Hexanol (r = 0.865, *p* < 0.05) and dihydro-β-ionone (r = 0.830, *p* < 0.05) showed significant positive correlations with soil AK content. Wang et al. also reported significant positive correlations between soil AK content and geraniol, α-ionone, and linalool [62]. Potassium can activate alcohol dehydrogenase in plants, promote conversion of carbohydrates into alcohols, and participate in energy metabolism related to terpene synthesis, thereby facilitating the formation of dihydro-β-ionone [70,71]. Furthermore, hexanol showed a significant positive correlation with soil OM content (r = 0.917, *p* < 0.05), likely due to the decomposition of OM providing carbon sources that serve as important precursors for hexanol synthesis. Dihydro-β-ionone (r = 0.587, *p* < 0.05) and p-menthatriene (r = 0.482, *p* < 0.05) also showed significant positive correlations with OM, indicating that OM facilitates synthesis of these terpene compounds. Consistent with these findings, Jiang et al. reported that sufficient soil OM supports microbial activity and provides slow-release resources, thereby favoring biosynthesis of terpenoids associated with floral aromas [61]. This effect may occur through provision of terpenoid precursors or through microbial processes that support precursor formation [72].

Additionally, AP showed significant positive correlations with p-menthatriene (r = 0.848), (E)-2-nonenal (r = 0.509), and butanal (r = 0.496) (*p* < 0.05), whereas a significant negative correlation was observed with benzaldehyde (r = −0.646, *p* < 0.05). These differences may arise from distinct formation pathways of aldehydes, as unsaturated aldehydes are mainly derived from lipid oxidation, whereas aromatic aldehydes are more closely associated with phenolic metabolism [73,74]. Bustamante et al. demonstrated that phosphorus is essential for terpenoid synthesis in *Rosmarinus officinalis* because it supports precursor generation and production of ATP and NADPH required for biosynthesis [75].

## 4. Conclusions

In this study, the volatile compounds of ‘Hujing Milu’ peaches from six regions were analyzed using combined HS-SPME-GC-MS and HS-GC-IMS approaches. Characteristic volatile compounds and their correlations with key soil factors were identified. Aldehydes, alcohols, and esters were the main volatile components of ‘Hujing Milu’ peaches, revealing significant regional differences in aroma type, proportion, and content. Notably, the highest total volatile content detected in the WX region. Based on OAV, ROAV, and VIP analyses, 17 volatile compounds were identified as potential biomarkers for distinguishing samples from the six regions. Characteristic volatile compounds were identified as follows: WX (hexanol, dihydro-β-ionone), FH (propyl butanoate, (E)-2-octenal), JY (benzaldehyde, ethyl acetate), MY (benzaldehyde), and FX (butanal). Correlation analysis indicated that contents of key volatile compounds, including hexanol, 2-methylbutanal, ethyl acetate, and dihydro-β-ionone, were significantly correlated with soil properties. Adjusting soil pH may enhance ester and aldehyde contents, whereas modifying soil OM may increase alcohol and terpene levels in peaches. Overall, this study clarifies regional aroma characteristics of ‘Hujing Milu’ peaches and their correlations with soil factors, providing theoretical support for origin tracing and aroma quality improvement.

## Figures and Tables

**Table 1:** Contents of volatile compounds in ‘Hujing Milu’ peaches from different regions detected by HS-SPME-GC-MS.

Category	Compounds	CAS	RT	RI ^a^	RI ^b^	|ΔRI|	Identification ^c^	Formula	Content (µg/kg)					
									JY	FX	FH	ZJG	WX	MY
Esters	γ-Decalactone	706-14-9	37.4	1470	1474	4	MS, RI, Std	C_10_H_18_O_2_	360.96 ± 44.75 c	1812.17 ± 320.3 a	634.45 ± 61.23 bc	836.08 ± 13.11 b	1818.26 ± 265.34 a	26.22 ± 14.76 d
	Dibutyl phthalate	84-74-2	51.2	1965	1966	1	MS, RI, Std	C_16_H_22_O_4_	9.99 ± 8.74 c	45.3 ± 9.2 ab	38.54 ± 6.68 abc	34.23 ± 9.03 abc	14.8 ± 14.07 bc	55.12 ± 33.53 a
	Hexyl acetate	142-92-7	20.7	1011	1011	0	MS, RI, Std	C_8_H_16_O_2_	1250.21 ± 167.12 c	585.53 ± 89.51 f	1748.23 ± 141.23 b	388.5 ± 90.37 f	2599.01 ± 67.29 a	982.43 ± 117.76 d
	γ-Dodecalactone	2305-05-7	43.6	1678	1686	8	MS, RI, Std	C_12_H_22_O_2_	ND	72.99 ± 6.35 b	41.97 ± 23.87 c	39.35 ± 2.04 c	184.57 ± 25.19 a	ND
	Methyl acetate	79-20-9	4.37	526	582	56	MS, RI, Std	C_3_H_6_O_2_	ND	1.25 ± 1.64	ND	ND	ND	ND
	δ-Decalactone	705-86-2	38.33	1496	1503	7	MS, RI, Std	C_10_H_18_O_2_	68.28 ± 4.15 d	671.04 ± 101.18 a	192.36 ± 9.2 c	220.47 ± 7.87 c	486.38 ± 113.27 b	ND
	Linalyl acetate	115-95-7	24.3	1257	1099	158	MS, RI, Std	C_12_H_20_O_2_	62.2 ± 53.9	ND	ND	ND	ND	ND
	Octyl acetate	112-14-1	28.43	1210	1208	2	MS, RI, Std	C_10_H_20_O_2_	ND	5.64 ± 1.46 b	ND	ND	9.02 ± 1.91 a	ND
	γ-Octalactone	104-50-7	30.35	1261	1262	1	MS, RI, Std	C_8_H_14_O_2_	24.82 ± 3.71 ab	75.56 ± 88.14 a	ND	28.35 ± 24.75 ab	52.69 ± 7.83 ab	ND
	Isopropyl myristate	110-27-0	47.35	1827	1821	6	MS, RI, Std	C_17_H_34_O_2_	ND	ND	ND	ND	ND	1.55 ± 1.76
	Ethyl acetate	141-78-6	5.81	612	623	11	MS, RI, Std	C_4_H_8_O_2_	36.85 ± 17.25 a	5.8 ± 1.01 b	ND	5.59 ± 1.61 b	ND	ND
	Nonyl acetate	143-13-5	31.96	1308	1307	1	MS, RI, Std	C_11_H_22_O_2_	ND	ND	ND	ND	15.41 ± 4.06	ND
	2-Ethyl hexyl salicylate	118-60-5	47.216	1811	1816	5	MS, RI, Std	C_15_H_22_O_3_	ND	4.27 ± 0.86 ab	6.75 ± 2.8 a	4.14 ± 2.48 ab	6.44 ± 1.69 a	5.09 ± 4.51 a
	L-Bornyl acetate	5655-61-8	31.4305	1284	1292	8	MS, RI, Std	C_12_H_20_O_2_	ND	ND	ND	ND	ND	7.35 ± 1.17
	(Z)-3-Hexen-1-yl propionate	33467-74-2	14.9034	1100	870	85	MS, RI, Std	C_9_H_16_O_2_	20.45 ± 27.08 bc	ND	54.71 ± 18.86 a	31.1 ± 16.27 ab	39.2 ± 8.87 ab	ND
	Linalyl butyrate	78-36-4	24.2815	1418	1099	319	MS, RI, Std	C_14_H_24_O_2_	58.98 ± 51.13	ND	ND	ND	ND	ND
Aldehydes	(E)-2-Nonenal	18829-56-6	26.6093	1162	1160	2	MS, RI, Std	C_9_H_16_O	48.64 ± 5.61 b	186.3 ± 12.04 a	198.11 ± 14.71 a	80.47 ± 18.36 b	182.04 ± 38.54 a	70.72 ± 12.11 b
	(E)-2-Octenal	2548-87-0	22.6273	1060	1058	2	MS, RI, Std	C_8_H_14_O	127.65 ± 15.54 c	302.73 ± 34.01 b	439.09 ± 62.4 a	266.3 ± 40.81 b	141.19 ± 23.63 c	95.69 ± 20.5 c
	2-Hexenal	505-57-7	14.1081	851	851	0	MS, RI, Std	C_6_H_10_O	10,575.29 ± 2948.54 a	4741.44 ± 3116.38 b	6864.2 ± 3384.58 ab	5634.9 ± 2163.59 b	3064.91 ± 1098.63 b	7295.89 ± 237.73 ab
	Citral	5392-40-5	30.7129	1276	1272	4	MS, RI, Std	C_10_H_16_O	13.29 ± 2.84 c	29.36 ± 2.07 b	46.15 ± 2.49 a	30.08 ± 4.15 b	27.28 ± 7.73 b	8.39 ± 2.67 c
	(E, Z)-2,6-Nonadienal	557-48-2	26.3791	1155	1154	1	MS, RI, Std	C_9_H_14_O	ND	ND	ND	ND	ND	14.9 ± 13.36
	(E, E)-2,4-Hexadienal	142-83-6	16.5305	911	910	1	MS, RI, Std	C_6_H_8_O	ND	ND	ND	ND	ND	228.84 ± 59.14
	Benzaldehyde	100-52-7	18.7225	962	962	0	MS, RI, Std	C_7_H_6_O	4872.24 ± 510.76 a	1258.78 ± 83.45 c	1793.24 ± 194.25 c	3079.27 ± 174.4 b	1846.97 ± 423.05 c	5110.02 ± 513.48 a
	(E)-2-Decenal	3913-81-3	30.3464	1263	1262	1	MS, RI, Std	C_10_H_18_O	ND	ND	64.82 ± 91.62a	ND	ND	13.86 ± 3.83 a
	Phenylacetaldehyde	122-78-1	22.1658	1045	1046	1	MS, RI, Std	C_8_H_8_O	4.11 ± 5.65	ND	ND	ND	ND	ND
	(E, E)-2,4-Nonadienal	5910-87-2	28.66	1216	1215	1	MS, RI, Std	C_9_H_14_O	ND	11.13 ± 10.44 b	32.58 ± 5.77 a	ND	ND	ND
	Nonanal	124-19-6	24.4516	1104	1103	1	MS, RI, Std	C_9_H_18_O	190.18 ± 15.23 c	345.24 ± 18.1 b	519.08 ± 85.11 a	348.26 ± 12.87 b	378.23 ± 113.84 b	ND
	2,4-Dimethylbenzaldehyde	15764-16-6	28.9276	1181	1222	41	MS, RI, Std	C_9_H_10_O	ND	ND	ND	120.93 ± 18.03 a	67.18 ± 10.58 b	50.13 ± 21.6 b
	β-Cyclocitral	432-25-7	29.1268	1220	1228	8	MS, RI, Std	C_10_H_16_O	ND	ND	31.42 ± 27.96	ND	ND	ND
	5-Methyl-2-thiophenecarboxaldehyde	13679-70-4	25.154	1118	1122	4	MS, RI, Std	C_6_H_6_OS	ND	ND	5.28 ± 4.02	ND	ND	ND
	(E, Z)-2,4-Decadienal	25152-83-4	31.5169	1295	1295	0	MS, RI, Std	C_10_H_16_O	ND	6.25 ± 5.43 ab	8.42 ± 7.45 a	ND	ND	ND
	Decanal	112-31-2	28.3011	1206	1205	1	MS, RI, Std	C_10_H_20_O	112.98 ± 2.79 b	189.19 ± 30.46 a	207.76 ± 13.76 a	105.41 ± 21.37 b	120.53 ± 22.19 b	ND
	(E, E)-2,4-Decadienal	25152-84-5	31.5191	1317	1295	22	MS, RI, Std	C_10_H_16_O	ND	ND	27.03 ± 19	ND	ND	ND
Alcohols	Linalool	78-70-6	24.3241	1099	1100	1	MS, RI, Std	C_10_H_18_O	ND	8641.94 ± 896.77 a	6139.84 ± 381.18 c	5964.65 ± 316.57 c	7529.31 ± 510.82 b	ND
	(E)-2-Nonen-1-ol	31502-14-4	24.4537	1176	1103	73	MS, RI, Std	C_9_H_18_O	ND	ND	ND	ND	ND	428.21 ± 190.57
	1,8-Cineole	470-82-6	21.7165	1032	1035	3	MS, RI, Std	C_10_H_18_O	ND	ND	ND	ND	ND	17.26 ± 3.32
	Hexanol	111-27-3	14.9775	868	872	4	MS, RI, Std	C_6_H_14_O	117.1 ± 102.87 b	176.83 ± 64.47 ab	174.03 ± 38.87 ab	102.86 ± 27.56 b	221.96 ± 30.62 a	ND
	(-)-α-Terpineol	10482-56-1	27.9479	1190	1195	5	MS, RI, Std	C_10_H_18_O	ND	120.88 ± 21.9 a	72.62 ± 7.37 b	107.91 ± 2.47 a	101.4 ± 17.14 a	ND
	Nonanol	143-08-8	26.9535	1173	1169	4	MS, RI, Std	C_9_H_20_O	ND	ND	ND	ND	26.38 ± 7.48	ND
	1-Octen-3-ol	3391-86-4	19.3374	980	977	3	MS, RI, Std	C_8_H_16_O	ND	ND	28.55 ± 24.83 ab	ND	36.7 ± 34.17 a	ND
Ketones	Methyl heptenone	110-93-0	19.7009	986	986	0	MS, RI, Std	C_8_H_14_O	93.97 ± 9.86 b	91.71 ± 100.74 b	280.88 ± 127.84 a	161.47 ± 68.74 ab	198.18 ± 54.66 ab	118.85 ± 14.76 b
	1-Hepten-3-one	2918-13-0	19.4	881	977	96	MS, RI, Std	C_7_H_12_O	40.78 ± 8.89 b	100.34 ± 56.16 ab	153.84 ± 92.84 a	77.64 ± 43.15 ab	44.77 ± 40.03 b	50.58 ± 9.82 b
	6-Pentyl-2H-pyran-2-one	27593-23-3	37.1969	1453	1467	14	MS, RI, Std	C_10_H_14_O_2_	37.21 ± 10.3 c	167.2 ± 23.12 a	84.08 ± 6.48 b	55.87 ± 2.52 bc	177.55 ± 43.83 a	ND
	(E)-Geranyl acetone	3796-70-1	36.7974	1453	1454	1	MS, RI, Std	C_13_H_22_O	70.77 ± 61.95 a	227.95 ± 197.88 a	332.98 ± 288.9 a	138.01 ± 121.01 a	287.08 ± 13.5 a	71.61 ± 18.78 a
	Neryl acetone	3879-26-3	36.7959	1435	1454	19	MS, RI, Std	C_13_H_22_O	73.89 ± 64.07	ND	ND	ND	ND	ND
	3-Octanone	106-68-3	19.6827	986	985	1	MS, RI, Std	C_8_H_16_O	ND	85.83 ± 30.24 a	ND	ND	36.13 ± 31.29 b	ND
	Dihydro-β-ionone	17283-81-7	36.5695	1433	1447	14	MS, RI, Std	C_13_H_22_O	209.51 ± 15.43 d	487.67 ± 29.51 c	724.02 ± 35.82 b	166.18 ± 0.14 d	986.5 ± 45.4 a	160.02 ± 37.39 d
	(E)-β-Ionone	79-77-6	38.0757	1486	1495	9	MS, RI, Std	C_13_H_20_O	ND	ND	ND	ND	124.28 ± 118.13	ND
	β-Damascenone	23726-93-4	34.8109	1386	1392	6	MS, RI, Std	C_13_H_18_O	3.21 ± 1 c	5.87 ± 0.35 b	9.52 ± 1.16 a	4.2 ± 0.48 c	4.14 ± 1.26 c	ND
	2-Octen-4-one	4643-27-0	20.8153	960	1013	53	MS, RI, Std	C_8_H_14_O	ND	ND	ND	ND	501.21 ± 452.18	ND
	α-Ionone	6901-97-9	36.2102	1429	1436	7	MS, RI, Std	C_13_H_20_O	ND	ND	6.96 ± 6.3	ND	ND	ND
Terpenes	3-Carene	13466-78-9	22.2341	1011	1048	37	MS, RI, Std	C_10_H_16_	ND	283.12 ± 49.79 a	157.74 ± 13.46 b	196.65 ± 12.6 b	187.07 ± 24.06 b	ND
	p-Cymene	99-87-6	24.7096	1025	1110	85	MS, RI, Std	C_10_H_14_	15.77 ± 14.21 b	333.13 ± 26.72 a	28.4 ± 29.33 b	305.28 ± 6.25 a	12.41 ± 12.47 b	ND
	Dextro-limonene	5989-27-5	21.5676	1018	1031	13	MS, RI, Std	C_10_H_16_	ND	316.64 ± 127.97 a	208.76 ± 22.58 a	254.14 ± 73.05 a	203.63 ± 37.78 a	ND
	α-Terpinene	99-86-5	21.0835	1017	1019	2	MS, RI, Std	C_10_H_16_	3.88 ± 4.75 c	80.07 ± 15.85 a	9.02 ± 7.83 bc	8.59 ± 7.73 bc	30.59 ± 21.15 b	ND
	(-)-β-Pinene	18172-67-3	19.8971	943	990	47	MS, RI, Std	C_10_H_16_	ND	ND	ND	385.91 ± 62.08 a	433.65 ± 82.7 a	ND
	α-Thujene	353313	22.7584	929	1061	132	MS, RI, Std	C_10_H_16_	ND	ND	19.64 ± 17.01 a	18.44 ± 16.8 ab	ND	ND
	Myrcene	123-35-3	19.9	991	992	1	MS, RI, Std	C_10_H_16_	ND	404.1 ± 111.08 a	215.53 ± 186.67 ab	128.09 ± 111.15 b	171.02 ± 148.24 b	ND
	Styrene	100-42-5	15.8069	893	892	1	MS, RI, Std	C_8_H_8_	61.67 ± 93.31 c	365.68 ± 43.53 a	ND	ND	ND	157.35 ± 15.04 b
	D-(+)-α-pinene	7785-70-8	17.6395	929	936	7	MS, RI, Std	C_10_H_16_	ND	ND	ND	ND	ND	7.64 ± 6.62
	Alloocimene	673-84-7	25.9414	1131	1142	11	MS, RI, Std	C_10_H_16_	ND	ND	ND	72.27 ± 62.59 a	24.72 ± 26.86 ab	ND
	p-Menthatriene	18368-95-1	25.5487	1119	1132	13	MS, RI, Std	C_10_H_14_	ND	74.89 ± 15.5 a	ND	47.52 ± 1.25 ab	41.39 ± 36.79 b	ND
Others	2-Ethyl furan	3208-16-0	8.6144	703	703	0	MS, RI, Std	C_6_H_8_O	56.55 ± 5.02 bcd	75.52 ± 9.9 abc	98.24 ± 10.47 a	79.79 ± 7.75 ab	46.08 ± 3.38 cd	42.64 ± 36.97 d
	2-Pentylfuran	3777-69-3	19.9167	993	991	2	MS, RI, Std	C_9_H_14_O	11.48 ± 17.74 b	16.51 ± 3.61 b	132.32 ± 130.59 a	41.26 ± 20.31 ab	30.55 ± 39.77 ab	18.02 ± 4.75 b
	Naphthalene	91-20-3	27.8449	1182	1192	10	MS, RI, Std	C_10_H_8_	72.67 ± 19.89 c	78.18 ± 5.17 bc	128.53 ± 17.93 a	87.04 ± 17.28 bc	107.16 ± 19.9 ab	81.09 ± 17.36 bc
	2-Methyl naphthalene	91-57-6	31.8652	1298	1305	7	MS, RI, Std	C_11_H_10_	12.95 ± 3.17 c	24.39 ± 4.12 b	33.68 ± 0.4 a	22.93 ± 1.35 b	21.8 ± 3.38 b	15.34 ± 4.23 c
	Valeric anhydride	2082-59-9	22.5272	1283	1055	227	MS, RI, Std	C_10_H_18_O_3_	106.37 ± 42.48 ab	99.36 ± 97.21 ab	191.29 ± 4.64 a	162.91 ± 141.2 a	106.48 ± 13.17 ab	ND
	Propionic anhydride	123-62-6	14.6666	921	866	55	MS, RI, Std	C_6_H_10_O_3_	ND	33.1 ± 29.27 a	ND	141.95 ± 226.7 a	ND	ND
	3-Butyl phthalide	6066-49-5	33.11	1656	1342	312	MS, RI, Std	C_12_H_14_O_2_	9.66 ± 1.94 c	21.08 ± 2.35 b	18.12 ± 0.21 b	20.26 ± 0.47 b	12.04 ± 1.14 c	28.59 ± 7.4 a
	Nonanoic acid	112-05-0	30.2885	1273	1260	13	MS, RI, Std	C_9_H_18_O_2_	ND	67.01 ± 76.63	ND	ND	ND	ND
	3-Methylvaleric acid	105-43-1	19.0773	947	971	24	MS, RI, Std	C_6_H_12_O_2_	ND	ND	ND	ND	25.27 ± 7.26	ND
	Theaspirane	36431-72-8	32.5898	1302	1326	24	MS, RI, Std	C_13_H_22_O	70.47 ± 7.77 e	282.29 ± 43.47 c	344.85 ± 23.26 b	88.72 ± 2.72 d e	420.71 ± 15.11 a	124.42 ± 22.23 d
	2-Methoxy-3-sec-butylpyrazine	24168-70-5	32.2666	1175	1317	142	MS, RI, Std	C_9_H_14_N_2_O	ND	ND	9.98 ± 12.79	ND	ND	ND

^a^ Literature RI, ^b^ Experiment RI, ^c^ MS, GC-mass spectrometry; RI, retention index and Std, confirmed by authentic standards; ND: not detected; Different letters represent significant differences (*p* < 0.05).

**Table 2:** Volatile compounds with OAV > 1 and VIP > 1 in ‘Hujing Milu’ peaches from six regions based on HS-SPME-GC-MS.

Compounds	Odor Threshold (μg/kg)	Odor Perception	OAV					
			JY	FX	FH	ZJG	WX	MY
(E)-2-Nonenal	0.19	Fatty, green, cucumber, citrus	255.98	980.51	1042.69	423.52	958.10	372.23
(E)-2-Octenal	3	Cucumber, fatty, banana, waxy, green	42.55	100.91	146.36	88.77	47.06	31.90
Benzaldehyde	750.89	Sharp, sweet, bitter almond, cherry	6.49	1.68	2.39	4.10	2.46	6.81
(E, E)-2,4-Nonadienal	0.1	Fatty, melon, waxy, green, violet	-	111.25	325.83	-	-	-
β-Cyclocitral	3	Herbal, rose, sweet, fruity	-	-	10.47	-	-	-
5-Methyl-2-thiophenecarboxaldehyde	1.75	Sweet, almond, cherry, woody	-	-	3.02	-	-	-
(E, Z)-2,4-Decadienal	0.04	Fried, fatty, green, waxy	-	156.27	210.58	-	-	-
(E, E)-2,4-Decadienal	0.027	Cucumber, melon, citrus, pumpkin, nut	-	-	1001.19	-	-	-
Dihydro-β-ionone	1	Earthy, woody, orris, amber	209.51	487.67	724.02	166.18	986.50	160.02
α-Ionone	3.78	floral	-	-	1.84	-	-	-
P-menthatriene	15	Turpentine, herbal, woody	-	4.99	-	3.17	2.76	-
2-Methoxy-3-sec-butylpyrazine	0.001	Green galbanum, bell, pepper	-	-	9982.24	-	-	-

Odor descriptions were obtained from the Perflavory Information System (https://www.perflavory.com, accessed on 7 November 2025).

**Table 3:** Volatile compounds detected in ‘Hujing Milu’ peaches from different regions using HS-GC-IMS.

Category	Compounds	RI	Rt (s)	Dt (a.u.)	Peak Volume					
					JY	FX	FH	ZJG	WX	MY
Esters	(Z)-3-Hexenyl acetate (M)	1006.9	260.472	1.32569	1292.69 ± 11.52 b	1181.75 ± 59.86 c	1298.65 ± 64.22 b	1245.26 ± 31.03 bc	1517 ± 29.53 a	736.94 ± 7.65 d
	(Z)-3-Hexenyl acetate (D)	1007.2	260.811	1.80945	826.87 ± 35.78 c	702.01 ± 70.16 d	927.13 ± 84.21 b	768.68 ± 54.18 cd	1491.81 ± 8.59 a	425.09 ± 2.72 e
	Hexyl acetate (M)	1015.6	268.629	1.38734	1132.5 ± 61.8 c	804.88 ± 37.61 d	1311.84 ± 59.01 b	641.12 ± 4.03 f	1988.28 ± 20.39 a	713.96 ± 16.65 e
	Hexyl acetate (D)	1015.9	268.969	1.89165	558.59 ± 56.01 c	318.06 ± 47.89 d	780.29 ± 88.83 b	228.92 ± 15.05 d	2386.17 ± 37.73 a	298.65 ± 8.85 d
	Methyl acetate (M)	558.2	72.618	1.19313	42.92 ± 3.18 cd	46.25 ± 2.25 cd	50.19 ± 4.15 c	81.72 ± 1.19 a	69.55 ± 11.23 b	39.33 ± 1.67 d
	Methyl acetate (D)	527.5	68.001	1.19679	111.28 ± 5.38 c	395.07 ± 81.6 b	97.82 ± 8.65 c	98.83 ± 3.81 c	523.67 ± 70.97 a	85.25 ± 14.87 c
	Propyl acetate (M)	700.0	99.843	1.1632	451.34 ± 12.04 a	123.64 ± 18.74 c	120.75 ± 3.78 c	76.73 ± 3.92 d	114.94 ± 15.96 c	152.25 ± 4.53 b
	Propyl acetate (D)	700.1	99.868	1.47729	118.25 ± 3.88 a	13.09 ± 1.86 b	12.76 ± 1.64 b	10.95 ± 0.44 b	11.93 ± 1.48 b	12.01 ± 0.97 b
	Ethyl acetate (M)	591.2	77.934	1.09609	838.98 ± 8.36 d	1111.74 ± 21.56 a	999.58 ± 10.94 bc	814.17 ± 11.84 d	947.03 ± 49.64 c	1016.98 ± 68.87 b
	Ethyl acetate (D)	588.9	77.547	1.33287	6822.95 ± 37.67 a	2053.97 ± 364.08 b	1229.6 ± 100.76 c	728.59 ± 34.08 d	726.86 ± 164.09 d	405.72 ± 86.19 e
	Propyl butanoate	885.8	180.039	1.26992	869.97 ± 18.97 b	665.96 ± 12.94 d	1070.53 ± 28.43 a	623.04 ± 27.53 e	709.55 ± 9.15 c	497.35 ± 18.81 f
	Isopentyl formate	777.7	130.378	1.26667	311.86 ± 7.23 c	334.45 ± 3.2 ab	325.64 ± 2.86 b	337.03 ± 4.17 a	289.33 ± 9.05 d	28.24 ± 4.7 e
	α-Angelica lactone	901.9	188.900	1.12502	119.12 ± 11.69 a	53 ± 3.61 d	69.73 ± 4.22 c	92.7 ± 9.13 b	51.62 ± 3.79 d	57.34 ± 3.13 d
Aldehydes	trans-2-Pentenal	741.5	115.14	1.35895	88.27 ± 4.45 a	65.64 ± 0.98 b	87.27 ± 8.7 a	50.46 ± 1.11 c	62.93 ± 2.17 b	95.54 ± 4.35 a
	Hexanal (M)	801.1	140.156	1.27025	2966.2 ± 40.11 b	2667.42 ± 18.3 d	3144.77 ± 13.46 a	2496.98 ± 2.32 e	2772.67 ± 31.56 c	2206.07 ± 43.97 f
	Hexanal (D)	800.3	139.804	1.55716	10,361.1 ± 146.29 b	9418.58 ± 50.45 d	11,518.45 ± 232.84 a	8832.96 ± 31.57 e	10,098.89 ± 69.28 c	8676.36 ± 59.8 e
	(E)-2-Hexenal	835.9	155.320	1.18101	682.4 ± 5.97 c	727.89 ± 13.06 b	662.65 ± 6.15 d	716.91 ± 6.26 b	750.61 ± 6.58 a	290.12 ± 12.69 e
	2-Hexenal(M)	853.2	163.524	1.17966	985.02 ± 12.58 c	1069.81 ± 3.53 a	924.35 ± 21.93 d	1090.89 ± 32.7 a	744.45 ± 8.57 e	560.21 ± 4.52 f
	2-Hexenal (D)	850.7	162.311	1.51631	11,316.25 ± 91.06 b	10,143.42 ± 150.1 c	11,762.12 ± 68.09 a	9742.43 ± 31.55 d	10,041.07 ± 106.41 c	11,467.09 ± 62.97 b
	Benzaldehyde (M)	958.6	224.047	1.15337	804.57 ± 41.51 a	351.66 ± 6.81 c	749.74 ± 38.79 a	441.77 ± 14.24 b	429.13 ± 23.1 b	394.28 ± 44.25 bc
	Benzaldehyde (D)	957.6	223.385	1.46472	221.23 ± 22.82 a	56.24 ± 1.2 bc	196.82 ± 25.65 a	75.41 ± 7.23 b	71.07 ± 6.39 b	40.29 ± 4.02 c
	2-Methylbutanal (M)	647.9	87.995	1.17418	1120.3 ± 11.97 a	956.56 ± 57.9 b	387.72 ± 25.87 c	1060.39 ± 21.22 a	909.12 ± 89.89 b	966.92 ± 6.64 b
	2-Methylbutanal (D)	643.8	87.226	1.39963	656.15 ± 14.35 b	237.92 ± 34.93 c	27.4 ± 1.42 d	290.13 ± 17.11 c	255.44 ± 64.78 c	1258.05 ± 28.23 a
	Butanal	583.2	76.609	1.28952	151.2 ± 4.32 bc	195.43 ± 8.17 a	168.14 ± 10.78 b	119.61 ± 1.12 d	151.08 ± 18.84 bc	142.2 ± 9.48 c
	(E)-2-Heptenal	951.2	219.121	1.25827	158.33 ± 1.36 c	178.15 ± 11.97 b	239.68 ± 7.04 a	133.28 ± 5.43 d	184.42 ± 6.91 b	93.57 ± 6.07 e
	Propanal	521.9	67.188	1.15551	130.3 ± 5.08 c	166.43 ± 13.66 b	100.29 ± 7.51 d	74.36 ± 2.2 e	297.34 ± 21.56 a	66.9 ± 7.36 e
	Pentanal (M)	686.1	95.509	1.19467	1137.6 ± 27.75 d	1367.11 ± 14.31 a	1305.25 ± 13.58 b	1152.59 ± 31.35 d	1261.66 ± 14.04 c	922.8 ± 22.55 e
	Pentanal (D)	686.5	95.59	1.42434	264.03 ± 25.19 c	321.02 ± 17.05 b	434.21 ± 49.16 a	176.61 ± 9.52 d	287.75 ± 5.23 bc	55.2 ± 2.05 e
Alcohols	Hexanol (M)	863.1	168.338	1.32669	559.38 ± 34.63 c	640.04 ± 23.03 b	532.71 ± 15.95 cd	673.4 ± 60.69 b	1212.24 ± 45.82 a	489.48 ± 2.81 d
	Hexanol (D)	865.5	169.537	1.64148	150.68 ± 13.1 c	158.52 ± 15.11 c	151.06 ± 11.67 c	164.62 ± 19.77 c	662.38 ± 12.83 a	198.59 ± 7.61 b
	(Z)-2-Penten-1-ol	758.4	121.99	1.60988	48.21 ± 4.28 bc	95.37 ± 35.25 a	32.21 ± 3.69 bc	49.14 ± 6.13 bc	59.15 ± 8.69 b	24.47 ± 1.66 c
	3-Methyl-1-butanol	760.9	123.063	1.23155	251.97 ± 5.27 b	401.05 ± 87.59 a	163 ± 24.79 c	250.52 ± 26.32 b	286.32 ± 20.32 b	88.59 ± 11.73 d
	2-Methylbutanol	743.4	115.875	1.22042	20.93 ± 0.84 b	21.01 ± 3.3 b	16.45 ± 1.07 b	18.77 ± 1.97 b	48.01 ± 5.19 a	20.68 ± 4.15 b
	2-Methyl-1-propanol	644.9	87.439	1.3679	235.47 ± 4.92 a	36.86 ± 5.06 c	17.29 ± 1.49 e	38.45 ± 1.93 c	26.19 ± 3.03 d	52.9 ± 0.76 b
	3-Heptanol	894.4	184.72	1.33702	267.61 ± 17.71 c	299.3 ± 7.15 b	299.65 ± 19.59 b	220.83 ± 5.04 d	341.18 ± 5.12 a	181.27 ± 2.55 e
	1-Pentanol	759.2	122.36	1.2719	272.96 ± 27.38 b	264.16 ± 30.93 b	450.23 ± 16.88 a	311.28 ± 64.25 b	257.33 ± 20.05 b	142.91 ± 7.62 c
	1-Propanol	570.7	74.578	1.24411	44.23 ± 2.69 b	45.6 ± 4.05 ab	38.78 ± 4.62 b	26.76 ± 1.61 c	52.69 ± 7.69 a	28.58 ± 1.89 c
	2-Propanethiol	587.6	77.332	1.15044	160.76 ± 10.53 c	169.57 ± 6.22 c	181.82 ± 5.1 c	350.09 ± 22.4 a	233.97 ± 8.48 b	131.88 ± 8.39 d
	2-Propanol	508.6	65.296	1.09736	210.08 ± 9.54 c	188.75 ± 8.02 c	193.54 ± 7.56 c	271.98 ± 7.3 b	422.7 ± 42.26 a	154.23 ± 9.93 d
	trans-2-Hexenol	870.9	172.263	1.1856	1376.39 ± 35.61 c	1145.2 ± 31.26 d	1433.42 ± 17.43 b	1112.06 ± 36.8 d	706.08 ± 24.81 e	1630.41 ± 13.32 a
	2-Octanol	1011.3	264.572	1.8516	212.44 ± 14.74 c	137.09 ± 12.08 d	261.19 ± 26.3 b	117.02 ± 3.91 de	537.77 ± 9.1 a	111.07 ± 4.13 e
	1-Penten-3-ol	685.2	95.332	1.34587	265.41 ± 11.23 b	205.04 ± 4.71 c	333.11 ± 22.27 a	211.85 ± 11.42 c	196.48 ± 33.27 c	133.35 ± 2.94 d
	5-Methyl-2-furanmethanol	954.2	221.095	1.57091	29.12 ± 4.48 bc	24.63 ± 1.76 bcd	43.85 ± 4.64 a	20.72 ± 1.61 d	30.58 ± 6.19 b	23.19 ± 0.62 cd
Ketones	2-Pentanone	686.7	95.629	1.38396	119.59 ± 6.47 d	148.88 ± 3.12 b	204 ± 22.39 a	126.62 ± 7.51 cd	141.04 ± 8.82 bc	47.65 ± 3.41 e
	2-Propanone	513	65.919	1.11768	208.98 ± 4.94 cd	208.13 ± 6.28 cd	270.08 ± 19.16 bc	286.28 ± 75.22 b	394.29 ± 55.37 a	183.01 ± 28.75 d
	3-Pentanone	684.2	95.106	1.10777	104.43 ± 3.79 b	95.96 ± 5.91 b	121.02 ± 4.59 a	125.78 ± 13.73 a	130.82 ± 8.69 a	67.41 ± 7.58 c
	6 methyl-5-hepten-2-one	986.9	243.949	1.17891	184.48 ± 14.31 b	162.46 ± 8.48 c	192.16 ± 7.53 b	127.96 ± 6.6 d	217.41 ± 11.44 a	141.44 ± 3.78 d
	1-Penten-3-one	689.3	96.257	1.08016	398.05 ± 29.3 a	221.73 ± 18.02 c	427.48 ± 25.1 a	311.13 ± 21.34 b	96.13 ± 6.5 d	188.71 ± 28.08 c
Others	Butanoic acid	786.2	134.113	1.39566	652.66 ± 26.33 bc	627.28 ± 13.24 cd	630.69 ± 15.85 cd	598.53 ± 6.68 d	672.6 ± 19 b	722.97 ± 18.63 a
	Propionic acid	741.3	115.05	1.10508	314.57 ± 6.68 a	252.21 ± 9.6 b	312.99 ± 15.1 a	183.07 ± 6.75 d	216.03 ± 5.51 c	211.94 ± 12.52 c
	1,2-Dimethoxyethane	669.2	92.101	1.3037	240.93 ± 37.18 a	63.39 ± 5.22 c	255.22 ± 19.22 a	114.28 ± 11.99 b	28.38 ± 1.27 d	78.27 ± 3.16 c
	2-Ethoxyethanol	718.6	106.416	1.09667	131.44 ± 4.07 a	70.06 ± 2.67 bc	66.73 ± 2.05 c	58.92 ± 2.05 d	73.69 ± 4.26 b	75.87 ± 3.36 b
	2-Methoxy-2-methylpropane	554.8	72.093	1.12656	249.42 ± 13.64 b	164.34 ± 12.69 c	139.57 ± 3.54 d	146.26 ± 5.83 d	169.31 ± 11.77 c	454.82 ± 6.88 a
	Tetrahydrofuran	612.2	81.529	1.06462	43.95 ± 1.75 e	144.31 ± 8.39 c	217.15 ± 6.55 a	149.62 ± 8.8 c	177.56 ± 6.52 b	93.28 ± 7.29 d
	Isoprene	518.2	66.649	1.22123	26.95 ± 4.8 cd	63.09 ± 21.17 a	32.23 ± 6.76 bc	9.83 ± 1.07 d	49.61 ± 9.39 ab	14.66 ± 0.61 cd
	2-Isopropyl-3-methoxy pyrazine	1102.5	365.659	1.22057	111.79 ± 8.53 e	716.86 ± 6.63 b	487.35 ± 23.67 d	535.62 ± 18.77 c	937.29 ± 5.91 a	128.8 ± 18.83 e

RI represents retention index; Rt represents retention time; Dt represents drift time; suffixes (D) and (M) represent dimer and monomer, respectively. Different letters indicate significant differences (*p* < 0.05).

**Table 4:** Volatile compounds with ROAV > 1 and VIP > 1 in ‘Hujing Milu’ peaches from six regions based on HS-GC-IMS.

Compounds	Odor Threshold (μg/kg)	Odor Perception	ROAV					
			JY	FX	FH	ZJG	WX	MY
Ethyl acetate	5	Fruity, sweet, weedy, green	57.49	26.19	15.20	13.62	13.00	12.79
Propyl butanoate	18	Fruity, sweet apricot, pineapple	1.81	1.53	2.03	1.53	1.53	1.24
2-Methylbutanal	1	Cocoa, coffee, nutty, malty, fatty	66.65	49.42	14.16	59.60	45.24	100.00
Butanal	2	Cocoa, malty, bready	2.84	4.04	2.87	2.64	2.93	3.20
Hexanol	5.6	Fruity, sweet, green	4.76	5.90	4.16	6.60	13.00	5.52

Odor descriptions were obtained from Perflavory Information System (https://www.perflavory.com, accessed on 9 November 2025).

**Table 5:** Characteristic volatile compounds of ‘Hujing Milu’ peaches from six regions based on HS-SPME-GC-MS and HS-GC-IMS.

Regions	Characteristic Compounds	Odor Perception
JY	Benzaldehyde	Sharp, sweet, bitter almond, cherry
	Hexanol	Earthy, woody, orris, amber
FX	(E, Z)-2,4-Decadienal	Fried, fatty, green, waxy
	(E, E)-2,4-Nonadienal	Fatty, melon, waxy, green, violet
	P-menthatriene	Turpentine, herbal, woody
	Butanal	Cocoa, malty, bready
FH	2-Methoxy-3-sec-butyl pyrazine	Green galbanum, bell, pepper
	(E)-2-Nonenal	Fatty, green, cucumber, citrus
	(E, E)-2,4-Decadienal	Cucumber, melon, citrus, pumpkin, nut
	Ethyl acetate	Fruity, sweet, weedy, green
	(E)-2-Octenal	Cucumber, fatty, banana, waxy, green
	β-Cyclocitral	Herbal, rose, sweet, fruity
	5-Methylthiophene-2-carboxaldehyde	Sweet, almond, cherry, woody
	(E, Z)-2,4-Decadienal	Fried, fatty, green, waxy
	α-Ionone	floral
ZJG	Propyl butanoate	Fruity, sweet apricot, pineapple
	P-menthatriene	Turpentine, herbal, woody
WX	Dihydro-β-ionone	Earthy, woody, orris, amber
	P-menthatriene	Turpentine, herbal, woody
MY	Benzaldehyde	Sharp, sweet, bitter almond, cherry
	2-Methylbutanal	Cocoa, coffee, nutty, malty, fatty

## Data Availability

The original contributions presented in this study are included in the article/Supplementary Material. Further inquiries can be directed to the corresponding author.

## Supplementary Materials

The following supporting information can be downloaded at: https://www.mdpi.com/article/10.3390/foods15061051/s1. Table S1: Odor activity values (OAV > 1) of ‘Hujing Milu’ peach samples from different regions. Figure S1: Five-fold cross-validation of the PLS-DA model based on HS-GC-SPME-MS. Table S2: Relative odor activity values (ROAV > 1) of ‘Hujing Milu’ peach samples from different regions. Figure S2: Five-fold cross-validation of the PLS-DA model based on HS-GC-IMS.
