# Supplementary material for: Comprehensive Analysis of Volatile Flavor Components in ‘Hujing Milu’ Peach from Different Regions Using HS-SPME-GC-MS and HS-GC-IMS
Source: Foods. 2026 Mar 17;15(6):1051. doi: 10.3390/foods15061051 (PMC13025913; doi:10.3390/foods15061051)
Supplement: Supplementary file 1 [file foods-15-01051-s001.zip › Table S1.pdf]

Table S1. Odor activity values (OAV >1) of 'Hujing Milu' peach samples from different regions.

Compounds	Odor threshold (μg/kg)	Odor perception	OAV					
			JY	FX	FH	ZJG	WX	MY
γ-Decalactone	1.1	Peach, coconut, buttery, sweet	328.15	1647.43	576.77	760.08	1652.97	23.84
Hexyl acetate	115	Fruity, green, apple, banana, sweet	10.87	5.09	15.20	3.38	22.60	8.54
γ-Dodecalactone	0.43	Fatty, peach, sweet, metallic, fruity	-	169.74	97.60	91.52	429.23	-
δ-Decalactone	66	Sweet, coconut, fruity, peach, creamy, dairy	1.03	10.17	2.91	3.34	7.37	-
γ-Octalactone	12	Sweet, coconut, waxy, creamy, dairy, fatty	2.07	6.30	-	2.36	4.39	-
Ethyl acetate	5	Fruity, sweet, weedy, green	7.37	1.16	-	1.12	-	-
(E)-2-Nonenal	0.19	Fatty, green, cucumber, citrus	255.98	980.51	1042.69	423.52	958.10	372.23
(E)-2-Octenal	3	Cucumber, fatty, banana, waxy, green	42.55	100.91	146.36	88.77	47.06	31.90
2-Hexenal	30	Sweet, almond, fruity, green, apple, plum, vegetable	352.51	158.05	228.81	187.83	102.16	243.20
Citral	28	Sharp, lemon, sweet	0.47	1.05	1.65	1.07	0.97	0.30
(E, Z)-2, 6-Nonadienal	0.8	Green, fatty, cucumber, violet	-	-	-	-	-	18.62
(E, E)-2, 4-Hexadienal	60	Sweet, green, spicy, floral, citrus	-	-	-	-	-	3.81
Benzaldehyde	750.89	Sharp, sweet, bitter almond, cherry	6.49	1.68	2.39	4.10	2.46	6.81
(E)-2-Decenal	0.3	Waxy, fatty, earthy, green, mushroom	-	-	216.06	-	-	46.20
Phenylacetaldehyde	4	Green, sweet, floral, honey, cocoa	1.03	-	-	-	-	-
(E, E)-2, 4-Nonadienal	0.1	Fatty, melon, waxy, green, violet	-	111.25	325.83	-	-	-
Nonanal	1.1	Rose, orris, orange, fatty	172.89	313.85	471.89	316.60	343.84	-
β-Cyclocitral	3	Herbal, rose, sweet, fruity	-	-	10.47	-	-	-

5-Methyl-2-thiophenecarboxaldehyde	1.75	Sweet, almond, cherry, woody	-	-	3.02	-	-	-
(E, Z)-2, 4-Decadienal	0.04	Fried, fatty, green, waxy	-	156.27	210.58	-	-	-
Decanal	3	Sweet, waxy, orange, citrus, floral	37.66	63.06	69.25	35.14	40.18	-
(E, E)-2, 4-Decadienal	0.027	Cucumber, melon, citrus, pumpkin, nut	-	-	1001.19	-	-	-
Linalool	0.22	Citrus, floral, sweet, rose, woody, green, blueberry	-	39281.54	27908.35	27112.06	34224.12	-
(E)-2-Nonen-1-ol	209	Waxy, green, violet, melon	-	-	-	-	-	2.05
1, 8-cineole	1.1	Eucalyptus, herbal	-	-	-	-	-	15.69
Hexanol	5.6	Fruity, alcoholic, sweet, green	20.91	31.58	31.08	18.37	39.63	-
1-Octen-3-ol	1.5	Mushroom, earthy, green	-	-	19.03	-	24.47	-
Methyl heptenone	68	Citrus, green, apple	1.38	1.35	4.13	2.37	2.91	1.75
1-Hepten-3-one	0.04	Metallic	1019.51	2508.45	3846.04	1940.90	1119.34	1264.43
6-Pentyl-2H-pyran-2-one	150	Lactonic, coconut, creamy, fatty, waxy	0.25	1.11	0.56	0.37	1.18	-
(E)-Geranyl acetone	60	Green, fruity, waxy, rose, woody	1.77	3.80	5.55	2.30	4.78	1.19
3-Octanone	21.4	Herbal, lavender, sweet, mushroom	-	4.01	-	-	1.69	-
Dihydro-β-ionone	1	Earthy, woody, orris, amber	209.51	487.67	724.02	166.18	986.50	160.02
(E)-β-ionone	0.007	Floral, woody, orris	-	-	-	-	17754.99	-
β-Damascenone	0.002	Apple, rose, honey, sweet	1603.47	2932.91	4761.13	2100.92	2071.69	-
α-Ionone	3.78	Floral	-	-	1.84	-	-	-
P-cymene	5.01	Citrus, woody	3.15	66.49	5.67	60.93	2.48	-
Dextro-limonene	34	Citrus, orange, sweet	-	9.31	6.14	7.47	5.99	-
α-Terpinene	80	Woody, lemon, herbal, citrus	0.05	1.00	0.11	0.11	0.38	-
Myrcene	1.2	Peppery, spicy, balsam, plastic	-	336.75	179.61	106.74	142.51	-
Styrene	65	Sweet, balsam, floral, plastic	0.95	5.63	-	-	-	2.42
D-(+)-α-pinene	2.2	Minty	-	-	-	-	-	3.47

P-menthatriene	15	Turpentine, herbal, woody	-	4.99	-	3.17	2.76	-
2-Pentylfuran	5.8	Fruity, green, earthy, beany, vegetable, metallic	1.98	2.85	22.81	7.11	5.27	3.11
Naphthalene	6	Pungent, tarry	12.11	13.03	21.42	14.51	17.86	13.52
2-Methyl naphthalene	3	Sweet, floral, woody	4.32	8.13	11.23	7.64	7.27	5.11
3-Butyl phthalide	10	Herbal, celery	0.97	2.11	1.81	2.03	1.20	2.86
2-Methoxy-3-sec-butylpyrazine	0.001	Green, galbanum, bell, pepper	-	-	9982.24	-	-	-

JY: Jianyang, Sichuan; FX: Fengxian, Shanghai; FH: Fenghua, Zhejiang; ZJG: Zhangjiagang, Jiangsu; WX: Wuxi, Jiangsu; MY: Mengyin, Shandong; Odor descriptions were reported at https://www.perflavory.com/search.php
