# Supplementary material for: Comprehensive Analysis of Volatile Flavor Components in ‘Hujing Milu’ Peach from Different Regions Using HS-SPME-GC-MS and HS-GC-IMS
Source: Foods. 2026 Mar 17;15(6):1051. doi: 10.3390/foods15061051 (PMC13025913; doi:10.3390/foods15061051)
Supplement: Supplementary file 1 [file foods-15-01051-s001.zip › Table S2.pdf]

Table S2. Relative odor activity value (ROAV >1) of 'Hujing Milu' peach samples from different regions.

Compounds	Odor threshold (μg/kg)	Odor perception	ROAV					
			JY	FX	FH	ZJG	WX	MY
(Z)-3-Hexenyl acetate	31	Fresh green, sweet, fruity, banana, apple, grassy	2.57	2.51	2.45	2.87	3.77	1.68
Hexyl acetate	115	Fruity, green apple, banana, sweet	0.55	0.40	0.62	0.33	1.48	0.40
Ethyl acetate	5	Fruity, sweet, weedy, green	57.49	26.19	15.20	13.62	13.00	12.79
Propyl butanoate	18	Fruity, sweet apricot, pineapple	1.81	1.53	2.03	1.53	1.53	1.24
Hexanal	5	Fresh green, fatty, grass, fruity	100.00	100.00	100.00	100.00	100.00	97.82
2-Hexenal	30	Sweet almond, fruity, green apple, vegetable	15.38	15.46	14.42	15.94	13.97	18.02
2-Methylbutanal	1	Cocoa, coffee, nutty, malty, fatty	66.65	49.42	14.16	59.60	45.24	100.00
Butanal	2	Cocoa, malty, bready	2.84	4.04	2.87	2.64	2.93	3.20
Pentanal	12	Fermented bready, fruity, nutty, berry	4.38	5.82	4.94	4.89	5.02	3.66
Hexanol	5.6	Fruity, sweet, green	4.76	5.90	4.16	6.60	13.00	5.52
3-Methyl-1-butanol	4	Whiskey, fruity, banana	2.36	4.15	1.39	2.76	2.78	1.00
2-Octanol	7.8	Green woody, herbal, earthy	1.02	0.73	1.14	0.66	2.68	0.64
2-Methoxy-2-methylpropane	7	Minty	1.34	0.97	0.68	0.92	0.94	2.92

JY: Jianyang, Sichuan; FX: Fengxian, Shanghai; FH: Fenghua, Zhejiang; ZJG: Zhangjiagang, Jiangsu; WX: Wuxi, Jiangsu; MY: Mengyin, Shandong; Odor descriptions were reported at https://www.perflavory.com/search.php
