# Supplementary figures and images for: Comprehensive Analysis of Volatile Flavor Components in ‘Hujing Milu’ Peach from Different Regions Using HS-SPME-GC-MS and HS-GC-IMS
Source: Foods. 2026 Mar 17;15(6):1051. doi: 10.3390/foods15061051 (PMC13025913; doi:10.3390/foods15061051)

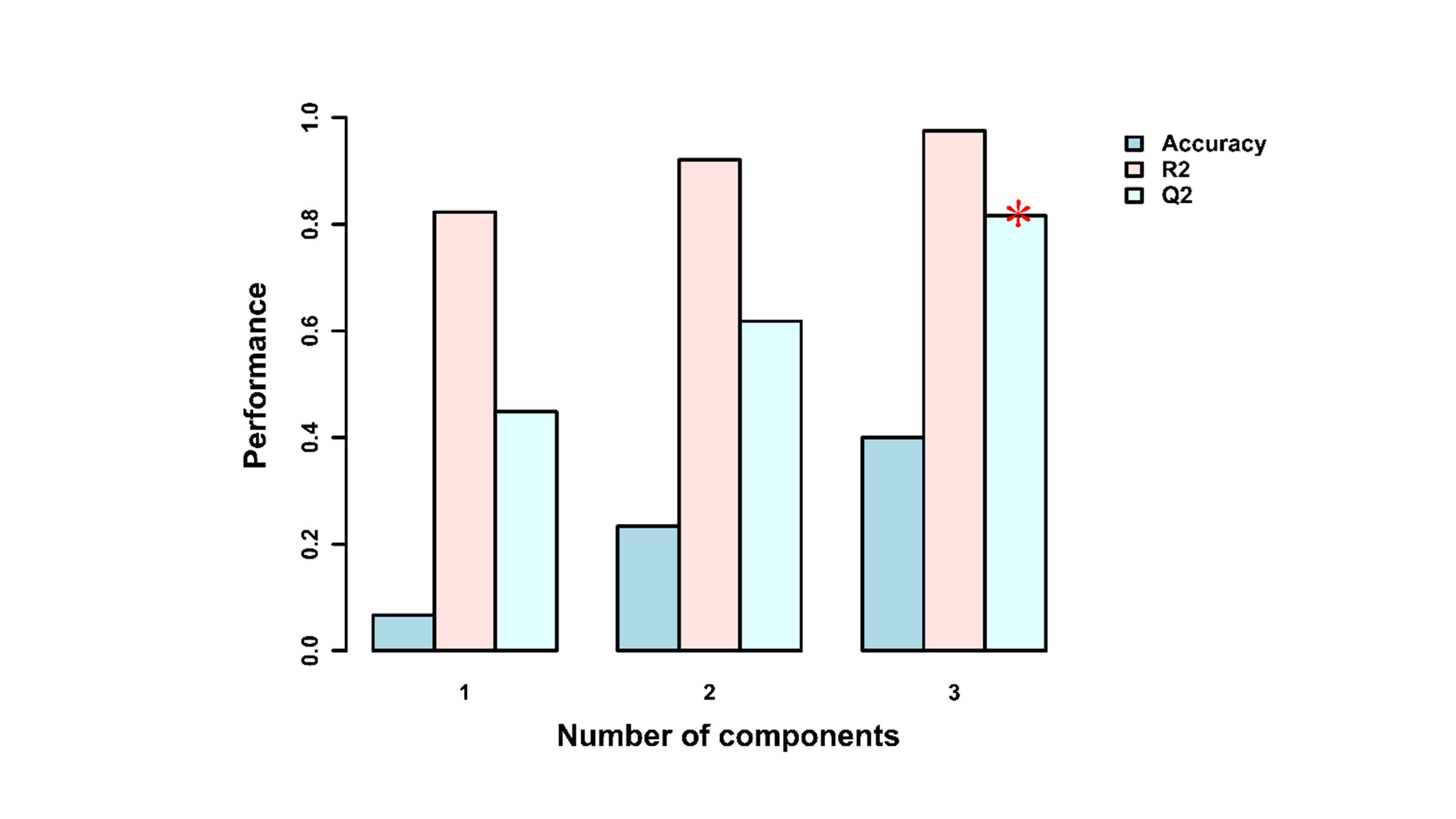

Supplement: Supplementary file 1 [file foods-15-01051-s001.zip › Figure S1.jpg]

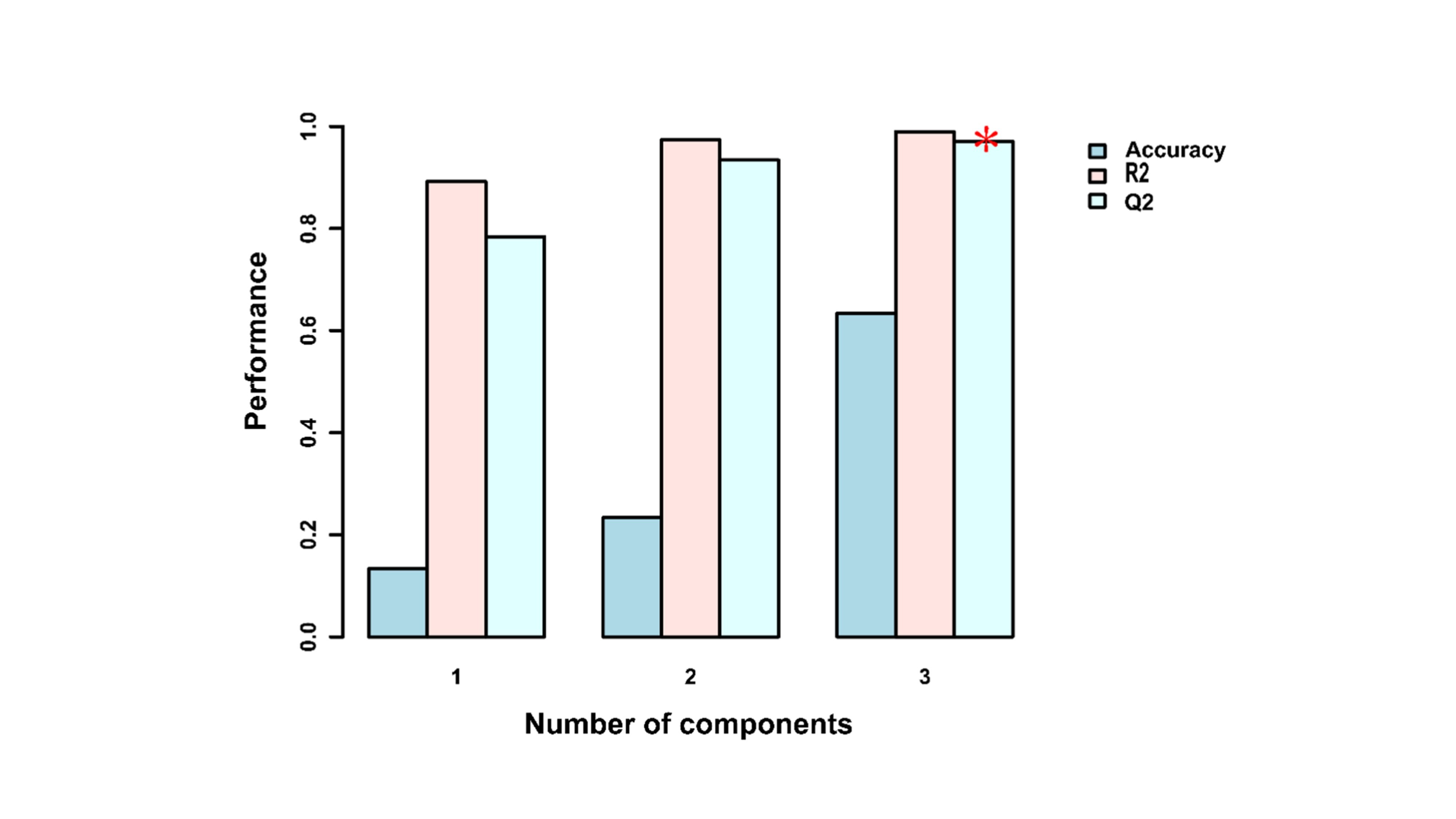

Supplement: Supplementary file 1 [file foods-15-01051-s001.zip › Figure S2.jpg]
